# Flattening filter‐free linac improves treatment delivery efficiency in stereotactic body radiation therapy

**DOI:** 10.1120/jacmp.v14i3.4126

**Published:** 2013-05-06

**Authors:** Brendan M. Prendergast, John B. Fiveash, Richard A. Popple, Grant M. Clark, Evan M. Thomas, Douglas J. Minnich, Rojymon Jacob, Sharon A. Spencer, James A. Bonner, Michael C. Dobelbower

**Affiliations:** ^1^ Department of Radiation Oncology University of Alabama at Birmingham Birmingham AL; ^2^ Department of Surgery, Division of Cardiothoracic Surgery University of Alabama at Birmingham Birmingham AL USA

**Keywords:** lung SBRT, liver SBRT, flattening filter‐free, treatment efficiency, treatment time

## Abstract

Stereotactic body radiation therapy (SBRT) employs precision target tracking and image‐guidance techniques to deliver ablative doses of radiation to localized malignancies; however, treatment with conventional photon beams requires lengthy treatment and immobilization times. The use of flattening filter‐free (FFF) beams operating at higher dose rates can shorten beam‐on time, and we hypothesize that it will shorten overall treatment delivery time. A total of 111 lung and liver SBRT cases treated at our institution from July 2008 to July 2011 were reviewed and 99 cases with complete data were identified. Treatment delivery times for cases treated with a FFF linac versus a conventional dose rate linac were compared. The frequency and type of intrafraction image guidance was also collected and compared between groups. Three hundred and ninety‐one individual SBRT fractions from 99 treatment plans were examined; 36 plans were treated with a FFF linac. In the FFF cohort, the mean (± standard deviation) treatment time (time elapsed from beam‐on until treatment end) and patient's immobilization time (time from first alignment image until treatment end) was 11.44 (± 6.3) and 21.08 (± 6.8) minutes compared to 32.94 (± 14.8) and 47.05 (± 17.6) minutes for the conventional cohort (p<0.01 for all values). Intrafraction‐computed tomography (CT) was used more often in the conventional cohort (84% vs. 25%; p<0.05), but use of orthogonal X‐ray imaging remained the same (16% vs. 19%). For lung and liver SBRT, a FFF linac reduces treatment and immobilization time by more than 50% compared to a conventional linac. In addition, treatment with a FFF linac is associated with less physician‐ordered image guidance, which contributes to further improvement in treatment delivery efficiency.

PACS number: 87.55.‐x

## I. INTRODUCTION

Stereotactic body radiation therapy (SBRT) is a method of delivering ablative doses of radiation to localized malignancies using concomitant image guidance and/or target tracking to ensure accurate target coverage with minimal margin. Based on successful early clinical results from phase I and II clinical trials, SBRT has become a common treatment strategy for small primary or metastatic lesions in the liver and lung.^1^, ^2^, ^3^, ^4^, ^5^, ^6^ One advantage of SBRT compared to conventionally fractionated therapy is a shortened treatment schedule, measured in days rather than weeks. Despite the benefit of an overall shorter treatment course, one disadvantage of SBRT is the lengthy time required to deliver each high‐dose treatment.

Prolonged treatment times are undesirable for patients who must remain immobilized in uncomfortable positions. Secondly, personal supervision of lengthy SBRT treatments is often an inefficient use of a practitioner's clinical time. Finally — and perhaps most importantly —prolonged treatment sessions may increase intrafraction motion due to patient discomfort in the treatment position, thereby jeopardizing the delicate therapeutic index of high‐dose radiation treatment.^(7)^

**Figure 1:**
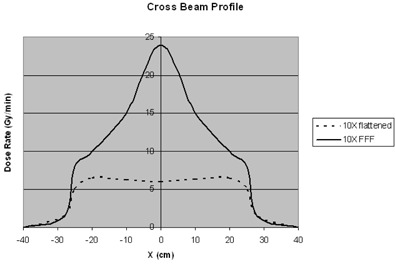
A schematic representation of the crossbeam profile of a conventional (with flattening filter) 10 megavolt photon beam (dashed line) is compared to the crossbeam profile of an unflattened photon beam (solid line) of equivalent energy. The unflattened beam has approximately four times higher dose rate at central axis.

Although several factors contribute to prolonged treatment times in SBRT, dose rate limitations of the linear accelerator (linac) are of paramount importance. Conventional linacs produce flattened photon beams at a dose rate of 400–600 monitor units per min (MU/min), but are capable of roughly four times higher dose rates with the flattening filter removed (Fig. 1). Existing preclinical data using phantom delivery confirm that linacs generating flattening filter‐free (FFF) photon beams can produce dosimetrically equivalent plans^(8)^ while reducing beam‐on time by more than 50%.^9^, ^10^ We hypothesized that clinical use of a FFF linac for SBRT would yield similar savings. In this study, we retrospectively compare beam‐on time, treatment time, and patient immobilization times for patients treated with a conventional linac using flattened photons versus a FFF linac for SBRT of lung and liver malignancies.

## II. MATERIALS AND METHODS

### A. Case selection

SBRT for lung and liver malignancies has been routinely performed at our institution since 2008. The medical records of 111 lung and liver SBRT patients treated from July 2008 to July 2011 were reviewed and 99 cases with full treatment records were selected for further study. For this analysis, SBRT was defined as radiotherapy delivered in 3–5 fractions of 6 Gy per fraction or higher, with concomitant image guidance and/or target motion tracking. Prior to September 2010, all patients were treated using a conventional linac (Varian IX and EX, Varian Medical Systems, Palo Alto, CA) producing flattened photons at 400–600 MU/min. In September 2010 we commissioned a FFF linac (Varian TrueBeam) capable of delivering 10 MV unflattened photons at a maximal dose rate of 2400 MU/min. SBRT cases performed after September 2010 were almost exclusively performed on this machine. Of the 99 included cases, 63 were treated with flattened photons at standard dose rates, and the remaining 36 were treated with unflattened beams at high‐dose rates. Dose schedule, treatment geometry, and treatment delivery technique were selected at the discretion of the treating oncologist.

### B. Immobilization and image guidance

At our institution, the standard immobilization procedure is a custom‐molded cradle (alpha cradle, Smithers Medical Products Inc, North Canton, OH) with an overhead arm device used to immobilize the patient's arms above the head; these devices were used for all patients in this study. All patients underwent standard pretreatment image guidance/target localization with paired orthogonal KV X‐rays matched to bony anatomy, followed by cone‐beam CT matched to soft tissue and bony anatomy. If significant patient motion (e.g., patient sits up, coughs violently, etc.) was seen on surveillance video, the standard pretreatment image sequence was reacquired. Cone‐beam CT alone was typically used to reaffirm target localization in between fields or arcs with substantial treatment times and was ordered at the discretion of the supervising oncologist. The same group of physicians and technicians treated patients with both treatment machines. An integrated record/verify software platform (Offline Review, Varian Medical Systems) was used to retrospectively assess the frequency and type of intrafraction imaging used during each fraction.

### C. Treatment efficiency measurements

We have identified three primary measures of treatment delivery efficiency: (1) beam‐on time (BOT), (2) treatment time (TxT), and (3) patient's immobilization time (IT). BOT was defined as the aggregate time in which the machine delivered photons for all fields or arcs in a given plan. TxT was defined as the time from onset of first treatment beam until completion of treatment, inclusive of any intrafraction imaging and/or shifts. IT begins at acquisition of the first pretreatment alignment image and ends at treatment completion; this time essentially recapitulates the length of time a patient is require to remain immobilized. Time parameters were collected for each SBRT fraction from an integrated treatment planning and record/verify software platform.

Additional treatment time parameters including pretreatment image guidance time (IGT) and intrafraction downtime (IFDT) were calculated as follows:

(1) IGT=IT‐TxT

(2) IFDT=TxT‐BOT

IGT was calculated to represent the amount of pretreatment time spent imaging and aligning the patient prior to beam‐on. IFDT was calculated to represent the time after treatment starts when the beam is paused for respiratory gating, imaging, or patient repositioning.

Lastly, observed clinical dose rate (CDR; measured in MU/minute) was calculated for the FFF cohort in order to compare the actual dose rate differences between the two methods. CDR is the number of MU required for a given plan divided by BOT.

### D. Statistics

Median and mean values for each of the six values described above were calculated for each SBRT fraction, and intergroup comparisons were made using t‐tests. Univariate regression analysis was performed to identify any factors associated with more efficient treatment delivery. MS Excel (Microsoft Corp, Redmond, WA) was used to construct and manage the database, and SPSS version 19 (IBM, Armonk, NY) was used for all statistical analysis.

## III. RESULTS

Ninety‐nine clinically delivered SBRT treatment plans, consisting of 391 individual fractions with available data, were reviewed. Seventy‐five patients were treated for primary tumors and 24 were treated for recurrent or metastatic disease. Treatment characteristics are reviewed in Table 1. Although a variety of fractionation schemes were employed, the median prescription was 12 Gy in 4 fractions (range: 6–20 Gy in 3–5 fractions), with the majority of treatments consisting of fractional doses greater than 10 Gy. Time‐intensive techniques such as respiratory gating and intensity‐modulated radiation therapy (IMRT) were used in a majority of cases (89% and 64%, respectively). FFF beams were used in 36% of cases. As stated in the Materials and Methods section above, these patients did not undergo a selection process for FFF treatment, as this technology was not available until September 2010.

**Figure 2:**
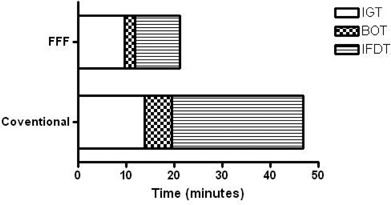
Treatment delivery time comparison: the overall length of the bars indicates the total immobilization time (IT) was 46.8 minutes and 21.1 minutes for the conventional and FFF cohorts, respectively. The unshaded region represents image guidance time (IGT), which was only marginally different between the two groups (13.9 vs. 9.6 minutes for conventional and FFF, respectively). The checkered portion represents beam‐on time (BOT), which was more than two times longer in the conventional group than the FFF group (5.6 vs. 2.3 minutes, respectively). The striped region represents intrafraction downtime (IFDT), which was substantially longer for the conventional group than the FFF group (27.3 minutes vs. 9.2 minutes, respectively).

Treatment with the FFF linac was significantly associated with higher mean CDR and shorter mean treatment time measurements (p<0.01 for all values; Table 2) compared to conventional SBRT. A graphical comparison of mean treatment time between the conventional and FFF cohorts is provided in Fig. 2. On univariate analysis, flattened beams, use of static fields, respiratory gating, and intrafraction imaging with cone‐beam CT were statistically associated (p<0.05) with prolonged TxT (Table 3). Intrafraction imaging occurred more frequently in patients treated at conventional dose rates, although this was due exclusively to the increased use of CT rather than orthogonal X‐ray imaging (Table 4).

**Table 1:** Patient and treatment characteristics for 99 patients treated with SBRT.

	*Overall*	*FFF*	*Conventional*
Disease site			
Thoracic	82	31	51
Hepatic	17	5	12
Dose schedule			
6 Gy x 5 fractions	3	1	2
8 Gy x 5 fractions	23	6	17
10 Gy x 5 fractions	12	7	5
12 Gy x 4 fractions	21	10	11
15 Gy x 3 fractions	16	5	11
20 Gy^a^ x 3 fractions	19	5	14
Other	5	2	3
Dose rate			
400–600 MU/min	63	0	63
2400 MU/min	36	36	0
IMRT			
Yes	64	34	30
No	35	2	33
Respiratory gating			
Yes	89	31	58
No	10	5	5
Treatment geometry			
Static gantry	67	9	58
Volumetric arc	32	27	5

^a^ Includes 18 Gy in 3 fractions with heterogeneity correction.

Gy: gray; MU/min: monitor units per minute.

**Table 2:** Treatment time components and observed dose rate.

*Time Component*	*FFF (minutes)*	*Conventional (minutes)*	*p‐value*
BOT	2.3±0.8	5.6±3.0	<0.01
TxT	11.5±6.3	32.9±14.7	<0.01
IT	21.1±6.7	46.8±17.6	<0.01
IGT	9.6±2.7	13.9±5.9	<0.01
IFDT	9.2±5.8	27.3±13.2	<0.01
CDR	1890±478	584±46	<0.01

Note: Mean time ± standard deviation (in minutes) of 5 treatment time components compared between FFF and conventional linac delivery. All tested time components were significantly shorter with FFF delivery. CDR (measured in MU/min) is significantly higher for FFF treatments.

BOT=beam‐on time; T×T=treatment time; IT=immobilization time; IGT=image guidance time; IFDT=intrafraction downtime; CDR=clinical dose rate; FFF=flattening filter‐free; MU/min=monitor units per minute.

**Table 3:** Univariate analysis of treatment time.

*Factor*	*Impact on TxT*	*p‐value*
FFF treatment	decreased	<0.01
VMAT	decreased	0.02
IMRT	none	NS
Respiratory gating	increased	<0.01
IF imaging		
X‐ray	none	NS
Cone‐beam CT	increased	<0.01
MU>3000	none	0.06

Note: Six factors tested for association with treatment time by univariate analysis. Use of FFF mode and VMAT were associated with shorter TxT; meanwhile, respiratory gating and intrafraction CT were associated with longer TxT. Use of IMRT, intrafraction X‐ray, and larger numbers of MUs had no significant effect on TxT.

FFF = flattening filter‐free; VMAT = volumetric‐modulated arc therapy; IMRT = intensity‐modulated radiation therapy; CT = computed tomography; MU = monitor unit.

**Table 4:** Frequency of intrafraction imaging use.

	*FFF*	*Conventional*	*p‐value*
Orthogonal X‐rays	19%	16%	NS
Cone‐beam CT	25%	84%	<0.01

Notes: Percentage of cases where intrafraction imaging with cone‐beam CT or X‐rays was used. CT was used three times more often in the conventional cohort compared to the FFF cohort. Orthogonal X‐rays were used in the same frequency in both cohorts.

FFF = flattening filter‐free; CT = computed tomography.

## IV. DISCUSSION

This institutional experience demonstrates that high‐dose‐rate SBRT treatments using a FFF linac are more efficient than similar treatments with a conventional dose rate linac. Furthermore, this study adds to the existing body of data supporting the feasibility of FFF beams for a variety of dose schedules and techniques (e.g., gating, IMRT, volumetric‐modulated arc therapy (VMAT)) in both palliative and definitive SBRT of the liver and lung.^(11)^ Preclinical studies have established the efficiency advantages of unflattened beams when delivered in phantom, and recent reports indicate these advantages are present in clinical applications;^(12)^ however, this is the first report comparing clinically treated cases delivered with flattened versus unflattened beams in a series of consecutive patients treated at the same institution.

A primary advantage of FFF beams is shortened dose delivery time. Until recently, it was unknown whether FFF mode would improve actual treatment delivery time in the clinical setting. We first reported that FFF linac‐based CNS radiosurgery produced improved treatment times compared to historical experience.^(13)^ However, it was not clear whether results from fixed‐target CNS lesions would translate to the setting of dynamic internal targets such as liver and lung malignancies. As expected, these results demonstrate that FFF mode decreases the BOT. Unexpectedly, the improvement in BOT was accompanied by dramatic improvements in overall treatment time. For instance, TxT — the time elapsed from first beam on until last beam off — was threefold shorter in the FFF group compared to the conventional cohort. Likewise, patients’ total immobilization time (IT) was reduced by more than half. Only a portion of this time savings could be directly accounted for by the shortened BOT; the remainder of the time savings came from a decrease in what we have called “intrafraction downtime” (IFDT), which was reduced from 27.3 to 9.2 minutes.

Although beam attenuation during gating and shifts contributes to IFDT, the primary determinant of IFDT is intrafraction imaging. In this study, intrafraction imaging was only ordered at the discretion of the treating physician if there was concern regarding intrafraction motion. As Table 4 demonstrates, there was no difference in the use of orthogonal X‐rays between the two groups. This is largely because X‐rays were typically used for initial alignment or realignment after significant motion events. On the other hand, CT was used three times more often in the conventional cohort than in the FFF cohort (25% vs. 84%), likely due to the assumption among supervising physicians that shorter treatments yield less intrafraction motion. Although intrafraction motion was not directly measured in this study, existing data support this assumption. In a cohort of 28 SBRT lung patients who underwent intrafraction CT, Purdie et al.^(14)^ and found that target motion was significantly increased when the interval from setup imaging to repeat imaging exceeded 34 minutes. In our FFF cohort, the median time from setup imaging to treatment completion (TxT) was less than 10 minutes; therefore, it is reasonable to expect that little intrafraction motion occurred. In summary, more efficient dose delivery with FFF beams shortens treatment time minimally by itself; however, the consequent reduction in use of intrafraction imaging leads to a dramatic reduction in total treatment time.

The pretreatment IGT was marginally different between the two groups (13 minutes for conventional vs. 9 minutes for FFF; p<0.01). Although one would not expect that a FFF linac would improve any time parameter when the treatment beam is off, the small yet statistically significant difference we observed in IGT was likely attributed to both improved on‐board imaging equipment with the FFF linac and improved staff proficiency with SBRT setup over time.

Although the nominal dose rate of FFF linac is 2400 MU/min, this is a potential maximum value that is not often achieved due to beam modulation or gantry rotation speed. In fact, our results indicate that machine output only reached 2400 MU/min in one case. However, the mean CDR was significantly higher in the FFF cohort (median 1890 MU/min), indicating that the dose‐rate advantage of FFF mode was realized in clinical SBRT applications. Use of VMAT in 75% of the FFF cohort accounted for most of the decrease in observed dose rate, as limited gantry rotational speed dictated machine output.

Some have suggested that the benefits of FFF treatment are not limited to dose‐rate improvements and subsequent efficiency advantages. Preclinical research suggests FFF beams generate less neutron contamination, lower doses outside the field edge, and less MLC leakage compared to beam‐flattened plans.^10^, ^15^, ^16^ Additional investigators suggest that the use of unflattened beams for IMRT could lead to lower rates of secondary malignancies given the observed 70% decrease in scattered photon dose.^(17)^ Further work will be necessary to determine the clinical significance of these above‐noted potential advantages for FFF beams.

Although interest in the clinical use of FFF photon beams dates back to 1991,^(18)^ their use has been limited to preclinical studies at research institutions until recently. With the recent commercial availability of linacs capable of operation without the flattening filter, clinical use for a broad variety of applications will likely become prevalent in coming years. Likewise, given the expanding use of IMRT in a variety of clinical applications, unflattened beams appear more practical. Because IMRT relies on modulation of beamlet fluence to achieve conformity and homogeneity, the flattening filter is superfluous in many common clinical scenarios.

Despite the robust data for 391 SBRT fractions presented here, we recognize the impact of several confounding variables when interpreting this data. For example, the FFF linac was able to perform respiratory‐gated volumetric arc therapy, which is expected to independently improve efficiency. Indeed, VMAT therapy was significantly associated with improved TxT and 27 of 32 VMAT plans were delivered on the FFF linac. Likewise, the FFF linac was capable of automatic field sequencing for static‐field IMRT, which reduces time between fields and is expected to independently improve efficiency. Although this represents the first published data comparing clinical treatment efficiency between conventional and high‐dose rates, interpretation is further limited by the retrospective nature of our data.

## V. CONCLUSIONS

The use of a FFF linac for SBRT of liver and lung malignancies is associated with substantial improvement in treatment delivery time. The reduction in beam‐on time with the FFF linac appears to impact the use of intrafraction imaging which, in turn, affects total treatment duration and patient immobilization time. Additional studies are warranted to evaluate the impact of shorter treatment times on the patient's treatment experience.

## ACKNOWLEDGMENTS

We thank Jason Schneider, RTT, for his assistance with data analysis and processing, and the remainder of our therapy staff for dedicated and compassionate treatment of our patients.
